# In vitro expansion affects the response of human bone marrow stromal cells to irradiation

**DOI:** 10.1186/s13287-019-1191-3

**Published:** 2019-03-08

**Authors:** Yang Xiang, Chun Wu, Jiang Wu, Weili Quan, Chao Cheng, Jian Zhou, Li Chen, Lixin Xiang, Fengjie Li, Kebin Zhang, Qian Ran, Yi Zhang, Zhongjun Li

**Affiliations:** 1 Department of Blood Transfusion, Irradiation biology laboratory, The Second Affiliated Hospital, Army Medical University, Xinqiao Road, Shapingba, Chongqing, 400037 China; 2 Central Laboratory, The Second Affiliated Hospital, Army Medical University, Xinqiao Road, Shapingba, Chongqing, 400037 China; 3 Center for Genome Analysis, ABLife Inc., Wuhan, 430075 Hubei China

**Keywords:** BMSCs, Irradiation, Cell passage, Transcriptome

## Abstract

**Background:**

Bone marrow stromal cells (BMSCs) are extensively used in regeneration therapy and cytology experiments simulate how BMSCs respond to radiation. Due to the small number and the heterogeneity of primary isolated BMSCs, extensive in vitro expansion is usually required before application, which affects the cellular characteristics and gene expression of BMSCs. However, whether the radiation response of BMSCs changes during in vitro expansion is unclear.

**Methods:**

In this study, BMSCs were passaged in vitro and irradiated at passage 6 (P6) and passage 10 (P10).

Then, apoptosis, the cell cycle, senescence, the cytokine secretion and the gene expression profile were analysed for the P6, P10, and non-irradiated (control) BMSCs at different post-irradiation time points.

**Results:**

The P6 BMSCs had a lower percentage of apoptotic cells than the P10 BMSCs at 24 and 48 h post-irradiation but not compared to that of the controls at 2 and 8 h post-irradiation.

The P6 BMSCs had a lower percentage of cells in S phase and a higher percentage in G1 phase than the P10 BMSCs at 2 and 8 h post-irradiation. The radiation had similar effects on the senescent cell level and impaired immunomodulation capacity of the P6 and P10 BMSCs. Regardless of whether they were irradiated, the P6 and P10 BMSCs always expressed a distinctive set of genes. The upregulated genes were enriched in pathways including the cell cycle, DNA replication and oocyte meiosis.

Then, a subset of conserved irradiation response genes across the BMSCs was identified, comprising 12 differentially upregulated genes and 5 differentially downregulated genes.

These genes were especially associated with the p53 signaling pathway, DNA damage and DNA repair. Furthermore, validation experiments revealed that the mRNA and protein levels of these conserved genes were different between the P6 and P10 BMSCs after irradiation. Weighted gene co-expression network analysis supported these findings and further revealed the effects of cell passage on the irradiation response in BMSCs.

**Conclusion:**

The results indicated that cell passage in vitro affected the irradiation response of BMSCs via molecular mechanisms that mediated differences in apoptosis, the cell cycle, senescence and the cytokine secretion. Thus, accurate cell passage information is not only important for transplantation therapy but also for future studies on the radiation response in BMSCs.

**Electronic supplementary material:**

The online version of this article (10.1186/s13287-019-1191-3) contains supplementary material, which is available to authorized users.

## Background

Ionizing radiation is ubiquitous and affects virtually everyone. All of us are exposed to doses of radiation from cosmic rays, soil radioactivity and diverse man-made electronic equipment to a certain extent [1]. In general, ionizing radiation can directly affect the cell by DNA oxidation and breakdown of double strands, which induce damage and variation of the chromosome [2]. Additionally, the water in the cell would be ionized to generate many reactive oxygen species (ROS), which will indirectly affect the cell by oxidizing the cell membrane. These adverse effects will kill the cell or induce cell canceration [2]. On the one hand, exposure to a relatively high dose of ionizing radiation will induce an acute response, including severe haematopoietic, gastrointestinal and cerebrovascular syndromes [3]. On the other hand, ionizing radiation is used to kill cancer cells, which will induce radiation resistance [4]. Thus, understanding the molecular mechanisms mediating the radiation response and resistance is urgent.

Bone marrow stromal cells (BMSCs) are important for recurring acute haematopoietic syndrome [5] due to their roles in maintaining the haematopoietic microenvironment [6]. As reported, BMSCs have relatively high resistance to radiation [7–9]. In fact, an early study showed that the cell cycle affected the radiation tolerance of BMSCs and that cells in S phase possessed the highest radiation resistance [10]. Moreover, BMSCs under differentiation commitments have various levels of radioresistance [11]. Regarding the molecular mechanism, BMSCs present radiation resistance by efficiently recognizing DNA damage, repairing double-strand breaks, clearing ROS and avoiding cell apoptosis [12]. For example, CRIF1 co-activates PKC-δ to phosphorylate NRF2, which decreases the high level of ROS level by elevating expression of antioxidant factors in BMSCs in response to radiation [13]. Thus, BMSCs are not only ideal regeneration therapy materials but are also a good model to study the radiation response and resistance.

Notably, BMSCs are bone marrow cells with an extremely low cell percentage (0.001–0.01%) [14] that decreases as the donor ages. Moreover, freshly isolated BMSCs are still highly heterogeneous [15, 16]. BMSCs must undergo extensive in vitro expansion prior to application in regeneration therapy and experiments. Therefore, determining whether in vitro expansion will affect the cellular characteristics of BMSCs is an important issue [17]. Previous studies have focused on the effects of in vitro expansion on the differentiation capability and senescence level in BMSCs, which may lead to negative results in clinical therapy [18–22]. However, whether in vitro expansion will affect the response of BMSCs to irradiation is unclear.

To answer this question, BMSCs were collected from passage 6 (P6) and passage 10 (P10) during in vitro expansion and irradiated with the same dose. The effects of irradiation on apoptosis, the cell cycle and the gene expression profiles of the P6 and P10 BMSCs were analysed. Our results showed that cell passage could affect the percentage of apoptotic cell and the cell cycle in BMSCs after irradiation. Moreover, irradiation had an inconsistent effect on the transcriptome profile of BMSCs at different cell passages. These results suggested that in vitro expansion affects the cellular characteristics and provide an important basis for further studies on the molecular mechanisms mediating the radiation response and resistance of BMSCs.

## Materials and methods

### Cell culture

To achieve a sufficient number of uniform cells for our experiments, BMSCs were purchased from ScienCell (Carlsbad, CA) three times on different dates and thus originated from independent cell cultures. The BMSCs were cultured in human mesenchymal stem cell growth medium (ScienCell, Carlsbad, CA) at 37 °C in a humidified atmosphere containing 5% CO_2_. BMSCs from the independent cell culture origins were used in the different experiments.

The purchased BMSCs were cultured for one passage from primary isolated bone marrow MSCs by ScienCell. Then, this lot of BMSCs was cultured for nine passages in our lab under the same conditions. Based on previous studies of the cellular characteristics of BMSCs during in vitro expansion [18, 23] and the cell number during passage in this study, passages 6 and 10 were used for the irradiation treatments (referred to as P6 and P10, respectively).

In brief, the BMSCs were replated into T75 culture flasks at the same initial density of 1 × 10^6^ cells/cm^2^. When cells became 80% confluent, they were harvested via trypsin digestion and counted using a cell counter (Countstar, Shanghai, China). The cultures and counts were maintained from P6 to P10. The population doubling (PD) and the doubling time (DT) for each passage were calculated based on the following equation:

PD = lnN_f_/N_i_/ ln 2DT = CT/PD

where Nf is the cell number at the final stage, Ni is the cell number at the initial seeding and CT is the cell culture time [24].

### Identification of BMSCs

First, we evaluated the typical surface markers of human BMSCs by flow cytometry as previously described [13]. The P6 and P10 BMSCs were characterized using fluorescent dye-conjugated antibodies targeting CD14, CD34, CD45, CD73, CD90, CD105 and HLA-DR. Isotype control antibodies were used as the negative control.

The differentiation capabilities of the P6 and P10 BMSCs were assessed using human mesenchymal stem cell osteogenic differentiation medium (catalog no. HUXMA-90021; Cyagen), human mesenchymal stem cell chondrogenic differentiation medium (catalog no. HUXMA-9004; Cyagen) and human mesenchymal stem cell adipogenic differentiation medium (catalog no. HUXMA-90031) [25]. The samples were fixed and stained with alizarin red S, Alcian blue and Oil Red O to evaluate osteogenic, chondrogenic and adipogenic differentiation, respectively.

### Irradiation of cells

**Fig. 1:**
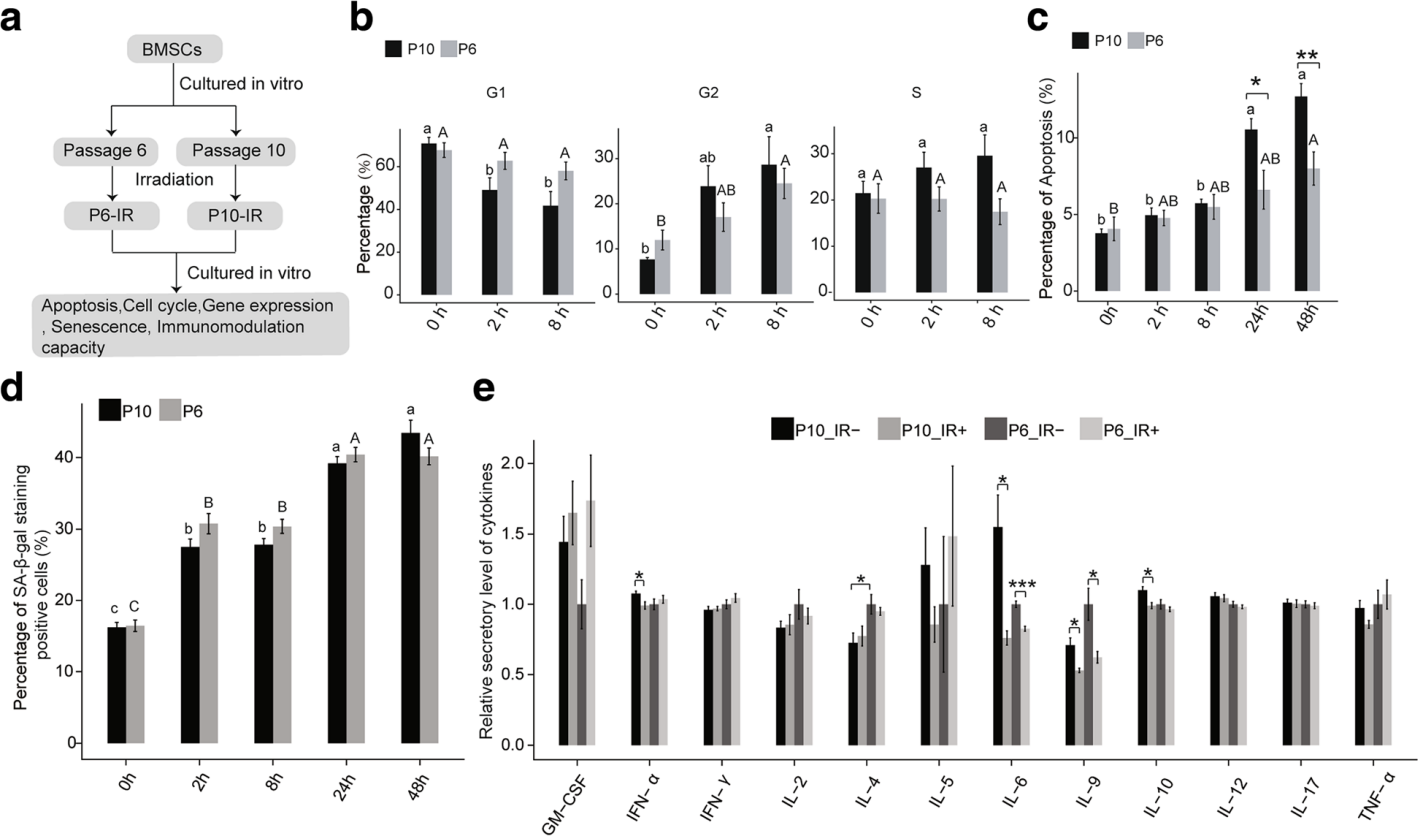
Cellular characteristics of BMSCs. **a** Pipeline of this work. **b** Cell cycle analysis of BMSCs from passages 6 and 10 of in vitro expansion. **c** Apoptosis analysis of BMSCs from passages 6 and 10 of in vitro expansion. **d** Senescence of BMSCs from passages 6 and 10 of in vitro expansion. **e** Cytokine secretory level of BMSCs from passages 6 and 10 of in vitro expansion with (IR+) or without (IR−) irradiation. Data are represented as the mean ± SEM. Student’s *t* test was performed to compare P6 and P10 BMSCs with significance set at a *P* value of less than 0.05. *P < 0.05, **P < 0.01. The same letter (lowercase for P10 and uppercase for P6, respectively) indicates no significant difference among different post-irradiation time (Tukey HSD, *P* < 0.05)

Due to differences in the tolerance to toxicity caused by race, only 7.7–9 Gy fractional total body irradiation is routinely used in the Chinese population before allogeneic haematopoietic stem cell transplantation (HSCT) [26]. To provide clues for the future clinical application of BMSCs, we used a relatively high radiation dose of 9 Gy. This radiation dose was also used in our previous studies on the biological effects of radiation in BMSCs [13, 25]. When the cultured P6 and P10 BMSCs reached 80% confluence, they were irradiated with 9 Gy by Co-60 at a dose rate of 700 cGy/min. The control group cells were placed in the same location but not exposed to irradiation. The BMSCs were sequentially cultured and were collected at 2 h and 8 h post-irradiation. According to our previous study [13], delay occurs in the emergence of apoptosis and senescence. Thus, some of cells were collected at 24 h and 48 h post-irradiation for the apoptosis analysis. The flow diagram of sample preparation is shown in Fig. 1a.

### Flow cytometric assays and senescence analysis

The BMSCs were collected and stained with the Annexin V-PE/7-AAD Apoptosis Detection kit. Apoptotic cells were detected using the Beckman MoFlo XDP, and the data was analysed with Flowjo (TreesStar) software. For the cell cycle analysis, the collected BMSCs were stained with propidium iodide. The cell cycle analysis was performed using the Beckman MoFlo XDP, and the data were analysed with ModFit (Verity Software House) software. Senescent cells were detected using a Senescence β-Galactosidase Staining Kit and HP1-γ staining [27].

### Detection of the immunomodulation capacity

The MACSplex Cytokin12 kit was used to evaluate the immunomodulation property. Granulocyte-stimulating factor (GM-CSF), interferon (IFN)-α, IFN-γ, interleukin (IL)-2, IL-4, IL-5, IL-6, IL-9, IL-10, IL-12p70, IL-17A and tumour necrosis factor (TNF)-α were detected by flow cytometry (MoFlo XDP, Beckman), according to the manufacturer’s instructions. The data were standardized to the secretory level of the irradiated P6 BMSCs.

### RNA extraction, library construction and sequencing

Total RNA was harvested from the BMSCs using the TRIzol reagent (Invitrogen) according to the manufacturer’s protocol. The total RNA was treated with the RQ1 DNase (Promega) to remove DNA. The quality and quantity of the purified RNA were determined by measuring the absorbance at 260 nm/280 nm (A260/A280) using the SmartsSpec Plus spectrophotometer (BioRad). The RNA integrity was further verified by 1.5% agarose gel electrophoresis. For each sample, 5 μg of total RNA was used for RNA-seq library preparation. Polyadenylated mRNAs were purified and concentrated with oligo(dT)-conjugated magnetic beads (Invitrogen). The purified mRNAs were fragmented at 95 °C, followed by end repair and 5′ adaptor ligation. Then, RNA reverse transcription was performed with an RT primer harbouring a 3′ adaptor sequence and randomized hexamers. The cDNAs were purified and amplified with the RNA-Seq Library Preparation Kit (Gnomegen). PCR products corresponding to 200–500 bps were purified, quantified and stored at − 80 °C before sequencing.

For high-throughput sequencing, the libraries were prepared following the manufacturer’s instructions and applied to the Illumina Nextseq 500 system for 151 nt paired-end sequencing by ABLife Inc. (Wuhan, China).

### RNA extraction and reverse transcription PCR

RNA extraction was performed with a commercial kit with the TRIzol® Reagent (Invitrogen). After RNA extraction, semi-quantitative RT-PCR was performed with the Superscript III One-Step RT-PCR System with platinum Taq High Fidelity (Invitrogen) using primer pairs specific for 150–250-base pair (bp) segments corresponding to the candidate genes. Quantitative real-time PCR was performed using the EXPRESS SYBR®GreenER™ qPCRSupermix Universal (Invitrogen) on the ABI Prism® 7900HT Sequence Detection System (Applied Biosystems). The experiments were repeated at least three times, and the statistical analysis was performed for individual experimental sets. All values were expressed as the threshold crossings (Ct). All PCR primer sequences can be found in the “Additional files” section (Additional file 1: Table S1).

### Western blotting analysis

BMSCs were analysed by western blotting based on our previous study [13]. For the preparation of total cell lysates, BMSCs were lysed in RIPA buffer (P0013B; Beyotime, Shanghai, China) at 4 °C. The samples were centrifuged, and the protein concentrations were checked using the Enhanced BCA Protein Assay kit (P0010S, Beyotime). The supernatants were separated on a 12% SDS-PAGE gel and subsequently transferred to a PVDF membrane (Immobilon-P Membrane, Millipore, USA). The membranes were blocked in 5% bovine serum albumin (BSA) for 1 h and then incubated with the appropriate primary antibodies overnight at 4 °C. Then, the membranes were incubated with the appropriate horseradish peroxidase-conjugated secondary antibody for 1 h at 24 °C. Immunoreactive bands were revealed by the BeyoECL Plus reagent (P0018, Beyotime) using the Photo-Image System (Molecular Dynamics, Sunnyvale, CA, USA). β-Actin was used as a loading control for the western blotting analysis. The following antibodies were used to analyse protein expression levels by western blotting: HJURP (ab224076, Abcam), GDF15 (ab39999, Abcam), CDKN1A (ab109520, Abcam) and p53 (ab1101, Abcam).

### Immunofluorescence analysis

After washing with PBS, the BMSCs were fixed in 4% formaldehyde. The cells were blocked in 5% BSA in PBS with 0.2% Triton X-100 for 10 min. The cells were incubated with primary antibodies (anti-HJURP, anti-GDF15, anti -CDKN1A and anti -p53) overnight at 4 °C. After washing with PBS, the cells were incubated with an appropriate secondary antibody in the dark at room temperature for 1 h. DAPI staining for DNA was performed, and images were obtained using a confocal LSM780 microscope (Carl Zeiss, Germany).

### Raw data cleaning and mapping statistics

First, the raw reads were discarded if the contained more than 2-N bases. Then, the reads were processed with the FASTX Toolkit (version 0.0.14) and cutadapt (version 1.7.1) to clip adaptor and remove sequences with low-quality bases and too-short reads (less than 16 nt). The clean reads were aligned to the human genome (GRCh38) by TopHat2 (two read mismatch and one seed mismatch). The reads were assigned to genes based on the gene annotation from GENCODE release 23 (https://www.gencodegenes.org/human/release_23.html). Aligned reads with more than one hit in the genome were discarded due to their ambiguous location. Uniquely mapped reads were used to calculate the expected fragments per kilobase of transcript per million fragments mapped (FPKM).

### Differentially expressed genes between two samples

Differentially expressed genes between the paired groups were analysed using edgeR [28] in the R packages. For each gene, a significant *P* value and false discovery rate (FDR) were obtained based on the negative binomial distribution model. Fold changes in gene expression were also estimated. The criteria for differentially expressed genes (DEGs) were set as a fold change > 1.5 or < 0.5 and a FDR < 0.05.

### KEGG enrichment analysis

Pathway analysis was performed using KOBAS (version 2.0) [29], based on the Kyoto Encyclopedia of Genes and Genomes (KEGG) database [30]. Pathways that were represented more significantly than would be expected due to random chance were identified using the KOBAS default method “h” by the Wallenius non-central hypergeometric distribution. The FDR (*q* value) was calculated based on the pathway analysis results using the Benjamini-Hochberg method by KOBAS. Then, the top 10 pathways with the lowest *q* values were plotted.

### Weighted gene co-expression network analysis

To obtain the mRNA expression modules, we applied weighted correlation network analysis (WGCNA) [31]. FPKM values were regarded as input. The output was the gene modules according to their expression patterns. For each gene module, an eigengene was chosen to represent the expression pattern. A heat map was plotted according to the gene modules.

### Statistical analysis

An unpaired two-tailed *t* test (between two groups) or analysis of variance (ANOVA) (multiple groups) with subsequent Tukey’s honestly significant difference (HSD) tests was performed for the cell biology and qPCR data. Probability (*P*) values < 0.05 were considered statistically significant. The data are presented as the mean ± standard error of mean (SEM). Each experiment was conducted in at least three biological replicates, except for the RNA_seq.

## Results

### There is no significant difference in the cell growth kinetics and the differentiation capability between the P6 and P10 BMSCs

The BMSCs exhibited similar morphology at every passage with spindle-shape morphology in our culture conditions (Additional file 2: Figure S1a). To clarify whether cell passage affects the cell growth, the population doubling (PD) and the doubling time (DT) for each passage (P6-P10) were calculated. The results showed that the PD numbers and the DT (in hour) for each passage gradually increased during in vitro expansion. However, the passage did not significantly affect PD numbers and DT (Additional file 2: Figure S1b, c) (ANOVA, *P* < 0.05).

To identity the cultured P6 and P10 BMSCs, typical surface markers of the human BMSCs were detected. The results showed the BMSCs-positive markers including the CD73, CD90 and CD105 were highly expressed both in the P6 and P10 BMSCs. Meanwhile, the cells were found to be negative for the CD14, CD34, CD45 and HLA-DR regardless of the cell passage (Additional file 3: Figure S2a). Then, the differentiation capability of the P6 and P10 BMSCs was evaluated. The results demonstrated that both the P6 and P10 BMSCs could differentiate into adipocytes, osteoblasts and chondrocytes. In addition, there were similar differentiations level between the P6 and P10 BMSCs (Additional file 3: Figure S2b). These results indicated in vitro expression did not significantly affect the cell growth kinetics and the differentiation capability of BMSCs from passages 6 to 10.

### Changes in apoptosis and the cell cycle but not the senescence and the cytokine secretion were sharper in the P10 than in the P6 BMSCs after irradiation

To evaluate the effects of BMSC passage on the response to irradiation, P6 and P10 BMSCs were collected for cell cycle and cell apoptosis analysis by FACS. The cell cycle analysis revealed that the proportions of cells in G1, G2 and S phases were similar between the P6 and P10 BMSCs (two-sample *t* test, *P* > 0.05). However, the compositions of the cells in the three phases were obviously different between the P6 and P10 BMSCs after irradiation, especially at 2 h and 8 h post-irradiation (Fig. 1b). After irradiation, the proportions of P6 BMSCs in G1 increased more robustly than the proportion in the P10 BMSCs, suggesting that the G1/S checkpoint responses to irradiation were more effective in the P6 BMSCs than in the P10 BMSCs (Fig. 1b). Notably, the proportions of cells in S phases did not change obviously in the P6 BMSCs after irradiation, whereas the proportion gradually increased in the P10 BMSCs (Fig. 1b). These results indicated that irradiation had more serious effects on the cell cycle in the P10 than in the P6 BMSCs, with more cells entering S and G2 phases.

For the apoptosis analysis, the post-radiation time, passage number and their interactions significantly affected the percentages of apoptotic in the BMSCs (ANOVA, *P* < 0.05). No significant differences on the proportion of apoptotic cells between the P6 and P10 BMSCs before irradiation and at 2 h and 8 h post-irradiation (two-sample *t* test, *P* > 0.05). However, the percentage of apoptotic P6 BMSCs was significantly lower than that of the P10 at 24 h and 48 h post-irradiation (Fig. 1c). The results showed that some cells would initiate the apoptosis programme after irradiation in both the P6 and P10 BMSCs, but the response was increased in the P10 BMSCs. Thus, both the cell cycle and apoptosis analyses showed that the P10 BMSCs were more sensitive to irradiation than the P6 BMSCs.

To confirm the senescent cell level in the P6 and P10 BMSCs with and without radiation, we assessed senescent cells using the Senescence β-Galactosidase Staining Kit and HP1-γ staining (Additional file 4: Figure S3). The results showed that irradiation increased the senescence populations both in the P6 and P10 BMSCs. However, no significant difference was observed between the P6 and P10 BMSCs with or without irradiation (Fig. 1d). These results indicate that irradiation had similar effects on the senescent cell level of the P6 and P10 BMSCs.

Finally, the impact of passage and irradiation on the immunomodulation capacity of BMSCs was evaluated using the secretion profile. The IL-4 level was decreased in the P10 BMSCs without IR, compared to those of the P6 BMSCS. No significant differences were observed in the other cytokines between the P6 and P10 BMSCs. In the P6 BMSCs, levels of the IL-6 and the IL-9 were suppressed by IR; the IFN-α, IL-6, IL-9 and IL-10 were suppressed by IR in the P10 BMSCs (Fig. 1e). Thus, these results indicate that the passage number only has a slight effect on the immunomodulation capacity of BMSCs. However, IR can lead to impaired immunomodulation capacity by decreasing the cytokine secretion levels.

### Gene expression profiles were significantly different between the P6 and P10 BMSCs before and after irradiation

To identify whether the gene expression profiles were different between the P6 and P10 BMSCs, RNA-seq was performed on the BMSCs before (sample name: P6_0h and P10_0h) and after irradiation (P6_2h, P6_8h, P10_2h and P10_8h). The obtained data and gene expression information was presented in the “Additional files” section (Additional file 5: Table S2; Additional file 6: Table S3; Additional file 7: Table S4).

**Fig. 2:**
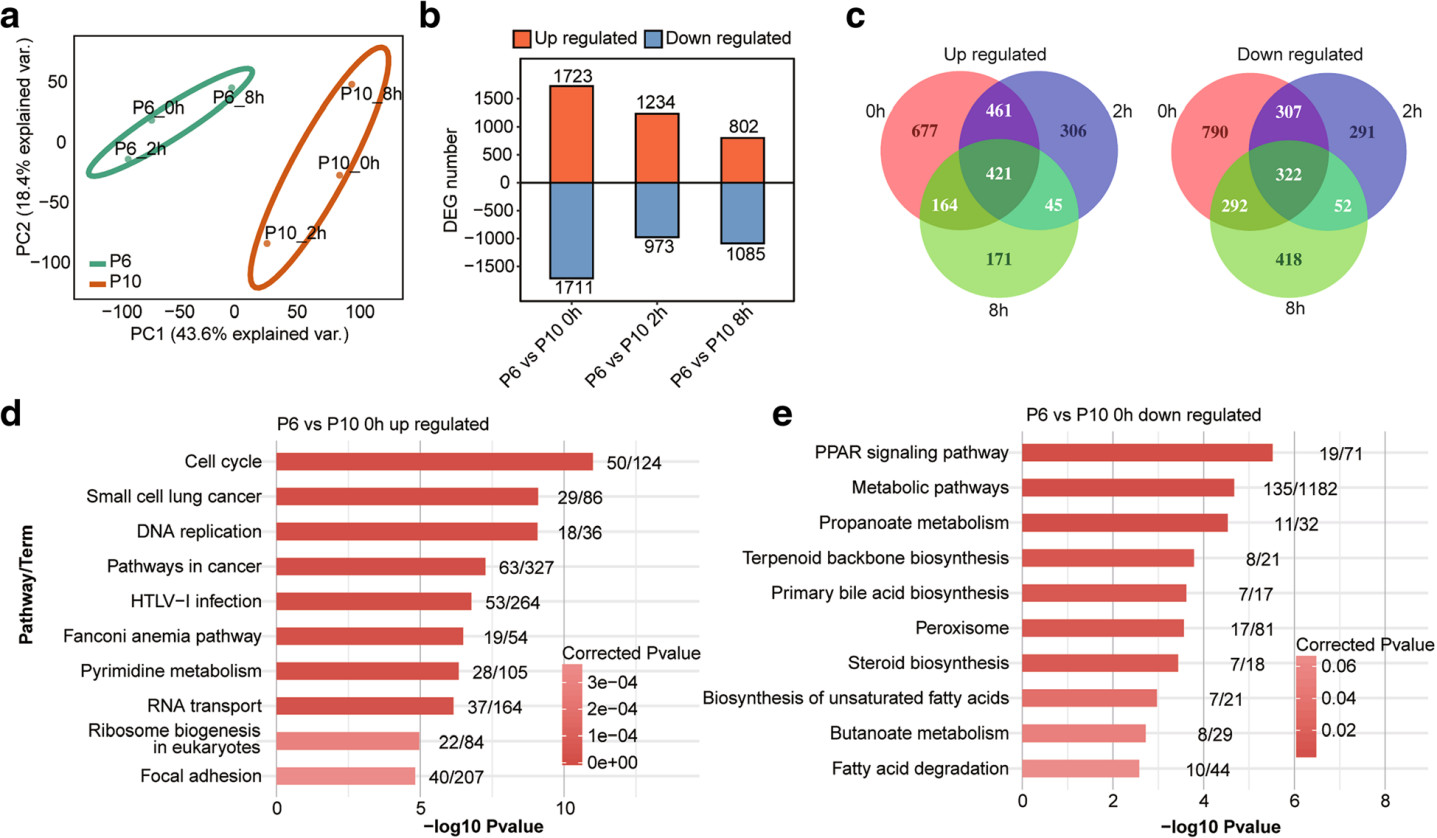
BMSC gene expression profiles. **a** PCA showing clustering by passage, with strong separation of the P6 and P10 BMSCs. **b** Bar plot illustrating up- and downregulated gene numbers from the P6 versus the P10 data sets. **c** Venn diagrams for up-(left) and downregulated (right) genes between the P6 and P10 BMSCs that were, both shared and had unique DEG numbers at the three time points. The top10 enriched KEGG pathways for up- (**d**) and downregulated (**e**) genes from the P6 versus P10 data sets at 0 h. The numbers after each bar indicate the detected genes (left) and the total background genes involved in the pathway

Unsupervised hierarchical clustering of the RNA-seq data showed a clear separation of the P6 and P10 BMSC samples (Additional file 8: Figure S4a), indicating obvious difference in their expression profiles. This finding was further supported by the principal component analysis (PCA) results, in which the P6 and P10 samples were obviously separated (Fig. 2a). Before irradiation, 3434 DEGs were detected; however, the numbers of DEGs decreased to 2207 and 1887 at 2 h and 8 h after irradiation, respectively (Fig. 2b; Additional file 9: Table S5). These results indicated that irradiation diminished the differences in the gene expression between the P6 and P10 BMSCs. However, some genes showed a distinct temporal-specific expression pattern between the P6 and P10 BMSCs (Fig. 2c). KEGG pathway analysis revealed that the upregulated genes (P6 vs. P10) were enriched in pathways related to the mitotic cell cycle, whereas the downregulated genes were enriched in metabolic and biosynthetic processes (Fig. 2d, e; Additional file 8: Figure S4b, c). Moreover, the co-upregulated DEGs between P6 and P10 both before and after irradiation were also enriched in the cell cycle, oocyte meiosis and DNA replication (Additional file 8: Figure S4d). The co-downregulated DEGs were enriched in pathways including steroid biosynthesis and metabolic pathways (Additional file 8: Figure S4d). These results showed different gene expression profiles between the P6 and P10 BMSCs.

### Conserved genes mediate the response to irradiation in the P6 and P10 BMSCs

**Fig. 3:**
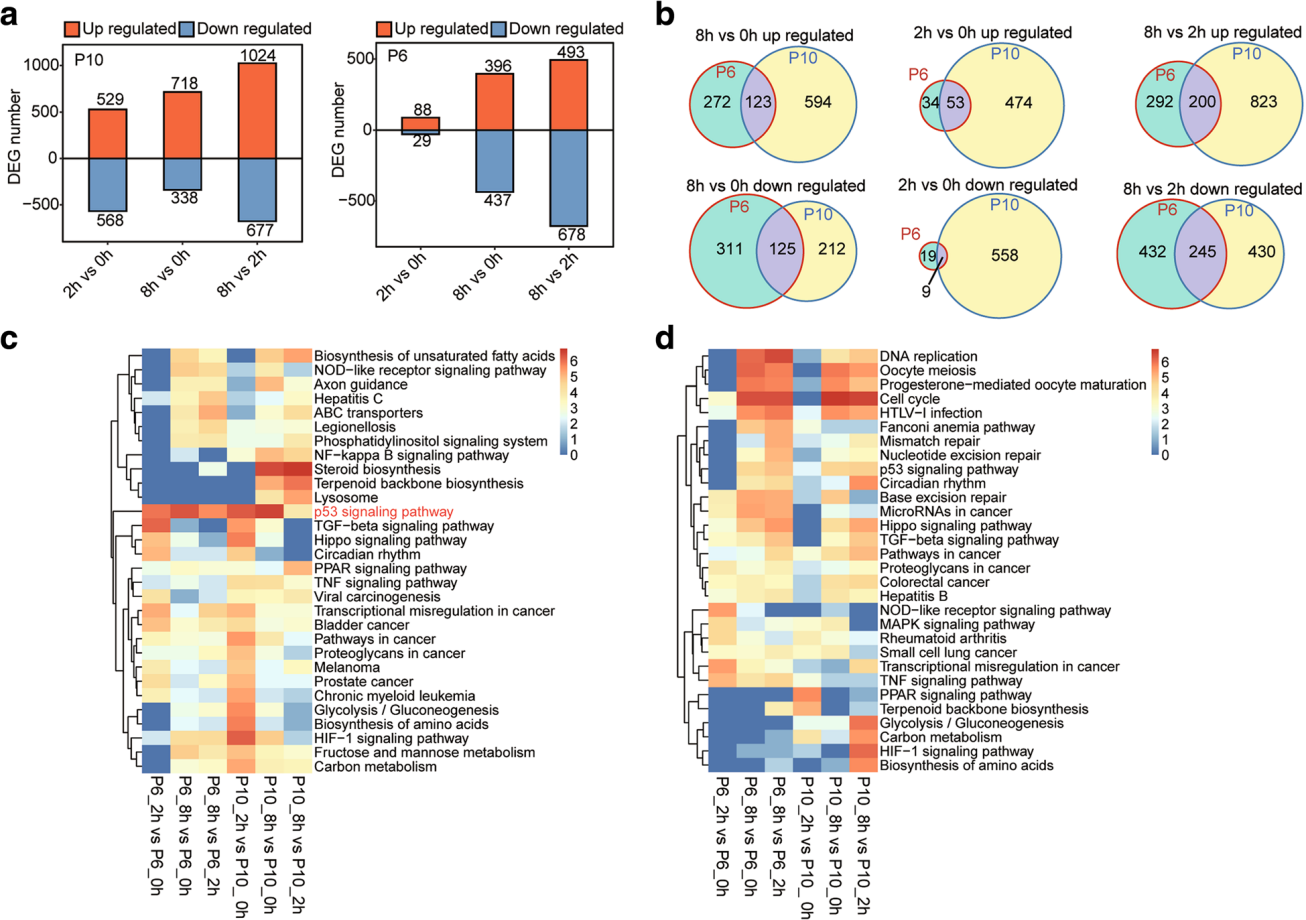
Molecular response of BMSCs to irradiation. **a** Bar plots representing up- and downregulated gene numbers between the different time points for the P6 (left) and P10 (right) BMSCs. **b** Venn diagrams of differentially expressed genes between the different time points illustrate shared and unique genes between the P6 and P10 BMSCs. The top 10 most enriched KEGG pathways were illustrated for genes that were up- (**c**) and downregulated (**d**) after irradiation. The colour scale shows the significance (*P* value) of the pathways

To identify the irradiation response genes in the BMSCs, we compared the expression profiles of both the P6 and P10 BMSCs after irradiation (2 h and 8 h) to those of the non-irradiated BMSCs (Additional file 10: Table S6). For the P10 BMSCs, a large number of genes were differentially expressed (P10_2h vs. P10_0h and P10_8h vs. P10_0h) after irradiation, but a relatively smaller number of genes presented different expression levels after irradiation in the P6 BMSCs, especially for P6_2h (117 DEGs) (Fig. 3a). However, many genes were significantly differentially expressed between the 2 h and 8 h time points after irradiation for both the P6 and P10 BMSCs (Fig. 3b), indicating a time-dependent irradiation response of BMSCs. Moreover, the number of overlapping DEGs between the non-irradiated BMSCs and both passages of BMSCs 2 h after irradiation was relatively small (Fig. 3b). To identify the functions of the DEGs after irradiation, KEGG pathway analysis was performed. The results showed that the upregulated genes were significantly enriched in the p53 signaling pathway (Fig. 3c) and the downregulated genes were enriched in pathways including the cell cycle and DNA replication (Fig. 3d). The upregulated overlapping DEGs between the P6 and P10 BMSCs were all enriched in the p53 signaling pathway for the three comparisons (2 h vs. 0 h, 8 h vs. 0 h and 8 h vs. 2 h) (Additional file 11: Figure S5a). The downregulated overlapping DEGs from two of the comparisons (8 h vs. 0 h and 8 h vs. 2 h but not the 2 h vs. 0 h comparison) were enriched in the cell cycle, oocyte meiosis and p53 signaling pathway (Additional file 11: Figure S5b). The expression profile of the P6 BMSCs showed almost no changes at 2 h after irradiation. In addition, the GO analysis showed that the upregulated genes were enriched in DNA damage response, small molecule metabolic process and apoptotic process. Conversely, the downregulated genes were enriched in terms including the cell cycle, DNA replication and DNA repair (Additional file 11: Figure S5c, d). These results indicated that both the P6 and P10 BMSCs would halt cell cycle and initiate DNA damage repair after irradiation.

### Validation of expression patterns for common response genes to irradiation in the P6 and P10 BMSCs

**Fig. 4:**
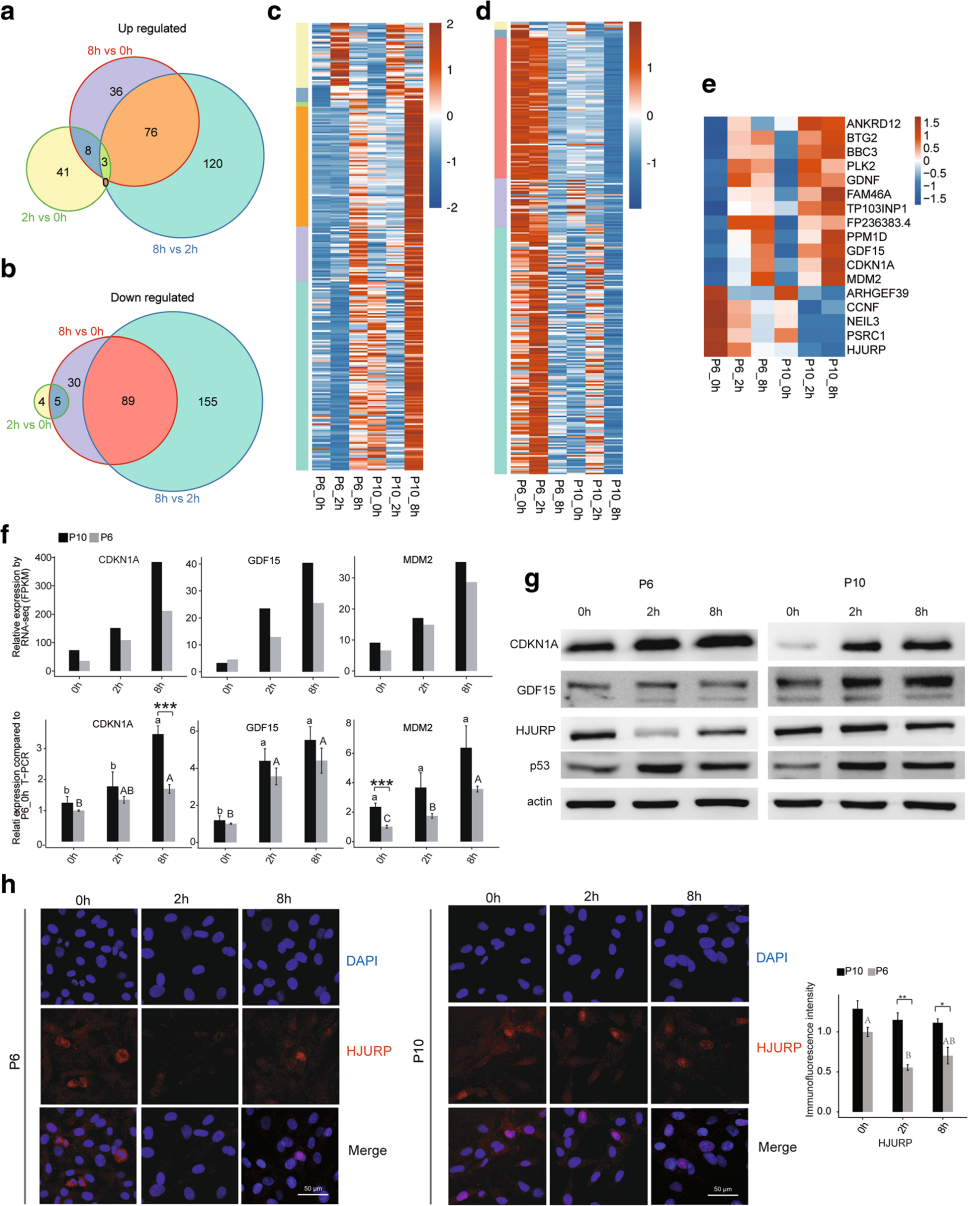
Expression patterns of conserved irradiation response genes in BMSCs. Venn diagrams for up- (**a**) and downregulated (**b**) genes shared by the P6 and P10 BMSCs according to the DEG analysis. **c** The expression pattern of the upregulated genes: each cluster of genes was distinguished accordingly by colour. **d** Expression patterns of the downregulated genes: each cluster of genes was distinguished accordingly by colour. **e** Expression pattern of genes that were either upregulated or downregulated at both 8 h and 2 h after irradiation relative to 0 h. **f** Relative expression levels of GDF15, CDKN1A and MDM2 measured by RNA-seq (FPKM) (up) and qRT-PCR (down). For qPCR, actin was used as the reference gene, and non-irradiated P6 BMSCs were used as the control groups. **g** Western blotting to evaluate CDKN1A, GDF15, HUJRP and p53 expression. All western blots are representative of three independent experiments. **h** Representative immunofluorescence staining in BMSCs. DAPI (blue), HJURP (red), merged images and quantification of immunofluorescence intensity (right) were shown. The photos were selected randomly. Scale bar 50 μm. Data are represented as the mean ± SEM. Student’s *t* test was performed to compare P6 and P10 BMSCs with significance set at a *P* value of less than 0.05. *P < 0.05, **P < 0.01. The same letter (lowercase for P10 and uppercase for P6, respectively) indicates no significant difference among different post-irradiation time (Tukey HSD, *P* < 0.05)

To further clarify the irradiation response of the BMSCs, the expression pattern of the overlapped DEGs between different comparisons from the two BMSC passages was analysed. In total, 200, 123 and 53 upregulated genes and 245, 125 and 9 downregulated genes overlapped in the three comparisons (2 h vs. 0 h, 8 h vs. 0 h and 8 h vs. 2 h, respectively) (Fig. 3b). Then, further analysis of these overlapped genes was conducted, and the results were presented with different colours (Fig. 4a, b). Moreover, the expression of genes from sections with different colours are shown (Fig. 4c, d). In detail, the genes from the different areas present various changes in expression after irradiation, but both the P6 and P10 BMSCs have similar expression patterns for DEGs from the same areas (Fig. 4c, d). In particular, the expression patterns of the up- or downregulated genes in both the P6 and P10 BMSCs at the two sampling time points (2 h and 8 h) after irradiation are shown (Fig. 4e). The changes in the mRNA level of these genes were validated by qPCR. The results showed that passage and irradiation significantly affect the expression of these genes in the BMSCs (Fig. 4f; Additional file 12: Figure S6). These genes were considered to represent conserved irradiation response genes in BMSCs.

To further validate the protein expression response to irradiation, three DEGs (CDKN1A, GDF15 and HJURP) were examined by western blotting and immunofluorescence. All three genes were important irradiation response genes [32, 33], and their mRNA levels were significantly changed in both the P6 and P10 BMSCs after irradiation (Fig. 2g). In addition, p53 protein expression was also evaluated due to important roles in the irradiation response. As shown in Fig. 4g, irradiation extensively increased CDKN1A, GDF15 and p53 expression in both P6 and P10 BMSCs. However, HJURP showed a differential expression pattern between the P6 and P10 BMSCs after irradiation (Fig. 4g; Additional file 13: Figure S7). The immunofluorescence analysis showed the same change pattern as the western blot for all four analysed genes (Fig. 4h and Additional file 13: Figure S7). These results indicated that some differences existed in the regulatory mechanisms that mediated the response to irradiation, although the response genes were conserved.

### The regulatory network mediating the response to irradiation is different between the P6 and P10 BMSCs

As shown in Fig. 2, the P6 BMSCs exhibited an initial expression profile that was distinct from that of the P10 BMSCs. To further estimate the effects of cell passage on the irradiated transcriptome in BMSCs, differentially expressed genes after irradiation (2 h vs. 0 h, 8 h vs. 0 h and 8 h vs. 2 h) in the P6 and P10 BMSCs were extracted to apply WGCNA analysis without a priori structural or functional knowledge.

**Fig. 5:**
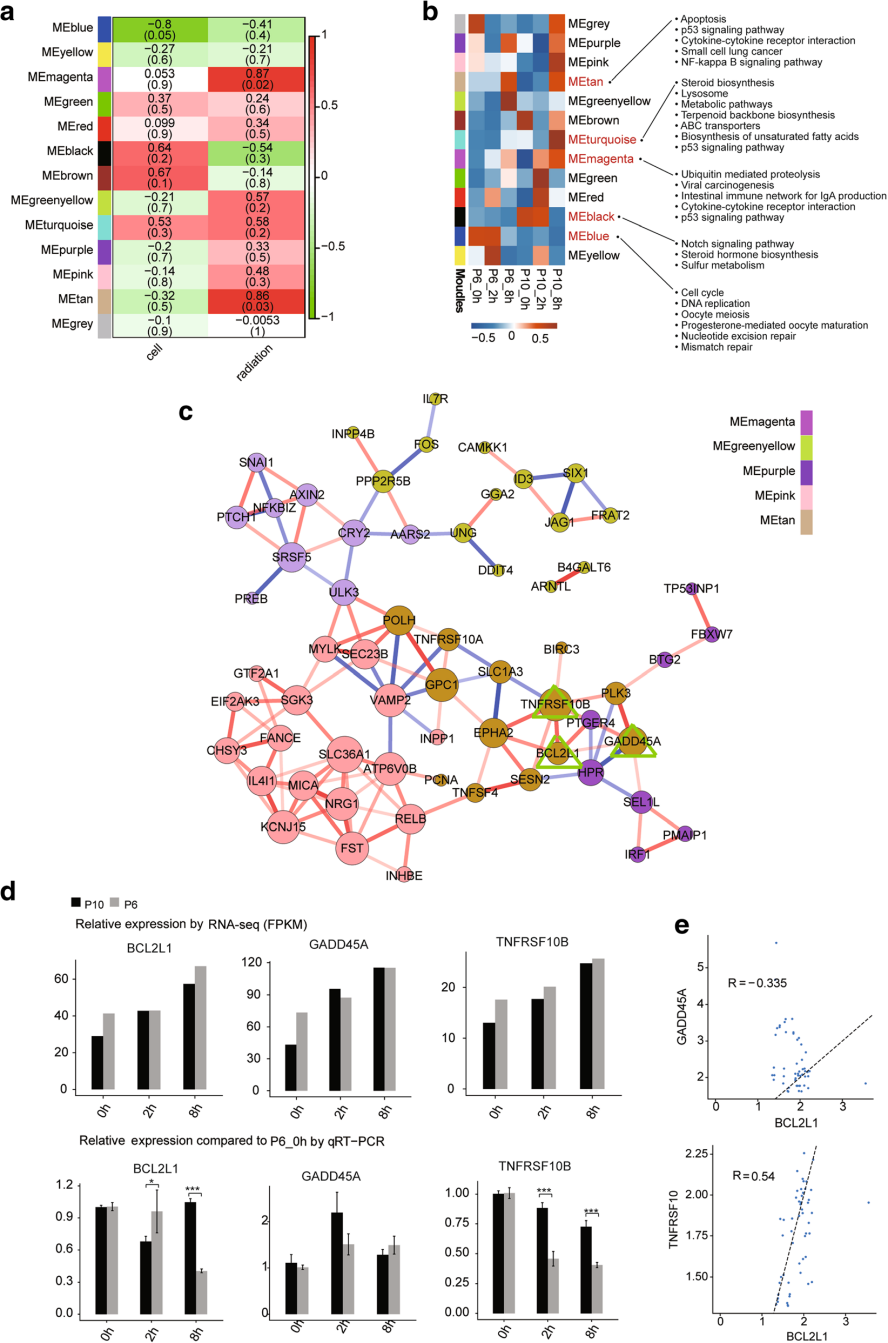
Weighted gene co-expression correlation network analysis of the differential expression genes. **a** Heat map showing the correlations of gene co-expressions modules (colour names) with the cell passage and radiation status. Numbers overlaying the heat map denote Pearson correlation coefficients (top number) and *P* values (lower number in brackets). Positive (negative) correlations indicate correlation with cell passages or the radiation treatment time background. **b** Heat map showing eigengene patterns of gene co-expression modules for six samples. The KEGG pathways on the right were significantly enriched for the genes assigned to the corresponding modules. **c** Sub-network of genes from five different gene co-expression modules. The node size indicates the correlation number. The red line represents positive correlations. The blue line represents negative correlation. **d** Relative expression levels of BCL2L1, TNFRSF10B and GADD45A measured by RNA-seq (FPKM) (up) and qRT-PCR (down). For qPCR, actin was used as the reference gene, and non-irradiated P6 BMSCs were used as the control groups; **e** Validation of expression correlations between two genes by qPCR. Data are represented as the mean ± SEM. Student’s *t* test was performed to compare P6 and P10 BMSCs with significance set at a *P* value of less than 0.05. *P < 0.05, **P < 0.01

By analysing our preprocessed P6 and P10 BMSCs, we identified 13 non-overlapping gene modules (arbitrarily labeled with colour names) (Fig. 5a). Genes from each co-expression modules still presented different expression patterns between the P6 and P10 BMSCs after irradiation (Additional file 14: Figure S8a). Moreover, the DEGs could be clustered specifically based on the modules (Additional file 14: Figure S8b). In addition, all 13 modules could be further clustered into three groups with similar gene expression patterns (Additional file 14: Figure S8c). Two modules (tan and magenta) were significantly correlated with radiation (*P* < 0.05) (Fig. 5a). Importantly, neither of these modules was correlated with the cell passage (Fig. 5a), further supporting the existence of a conserved response to radiation in different BMSC cell passages. As shown in Fig. 5a, only one module (blue) was significantly inversely correlated with cell passage (*P* < 0.05), whereas two modules (black and brown) were potentially positively correlated, with relatively high Pearson correlation coefficients. Although one module (turquoise) was highly correlated with both the cell passage and irradiation, the *P* values did not indicate a significant correlation (Fig. 5a).

The expression patterns of genes for each module are illustrated (Fig. 5b). The KEGG pathway analysis showed that the genes from both the tan and magenta module were most significantly enriched in pathways including apoptosis, the p53 signaling pathway and cytokine-cytokine receptor interactions (Fig. 5b). The genes in the blue module were enriched in the cell cycle, DNA replication and oocyte meiosis. Importantly, many of the genes in the turquoise module were also enriched in the p53 signaling pathway, but the expression patterns were different between the P6 and P10 BMSCs after irradiation. The full listings of the KEGG terms enriched in all five modules are provided in the “Additional files” section (Additional file 15: Table S7). The sub-network of genes from five different gene co-expression modules (magenta, greenyellow, purple, pink, tan) were further analysed (Fig. 5c). One of the most important nodes was Gadd45a, which was sustainably upregulated in both the P6 and P10 BMSCs after irradiation. Then, the mRNA expression levels of Gadd45a, Bcl2l1 and Tnfrsf10b with direct correlation in the network were validated by qPCR (Fig. 5d). The results showed that a weak positive correlation existed between the mRNA expression levels of Tnfrsf10b and that of Bcl2l1 (Fig. 5e).

In addition, the expression levels of genes from the turquoise module were decreased at 2 h, but increased at 8 h in both the P6 and P10 BMSCs. After irradiation, the expression levels of genes from the black module were decreased in the P6 BMSCs, but increased in the P10 BMSCs. Interestingly, the genes from the blue module had higher expression level in the P6 BMSCs than in the P10 BMSCs, but the expression level was decreased in both passages of BMSCs after irradiation. The relationships between the genes from the three modules were further displayed using module plots (Additional file 14: Figure S5d, e, f). These results further demonstrated that cell passage affected the response to irradiation in BMSCs.

## Discussion

Although BMSCs play an important role in regenerative medicine and stem cell research, they are a very rare population and extensive in vitro expansion is required [17]. Changes in the gene expression profile and cellular characteristics, including the telomere length, division activity and differentiation potential, during in vitro cell passage, have been studied [18, 22, 34]. Here, we investigated whether cell passage affected the response to irradiation by analysing the BMSC cell cycle, apoptosis, senescence, immunomodulation capacity and transcriptome in BMSCs.

In our study, phenotypic characteristics including apoptosis and the cell cycle are similar between the P6 and P10 BMSCs without irradiation, which was consistent with the results of a previous study on different cell passages [18]. Notably, obvious changes in senescence and apoptosis were observed in both the P6 and P10 BMSCs after irradiation. A significant difference in apoptosis but not senescence was observed between the P6 and P10 BMSCs after irradiation. However, BMSCs were shown to be prone to senescence rather than apoptosis after exposure to a low radiation dose in previous studies [35, 36]. Generally, BMSCs at late passages should be more resistant to apoptosis since they should have a higher percentage of senescent cells. We speculate that radiation dose, irradiation type and post-irradiation time selection will affect the investigation of these cell biology characteristics.

In fact, transcriptome analysis results showed that the P6 BMSCs were obviously different from the P10 BMSCs. GO analysis of DEGs between the two cell passages showed significant signs of senescence or ageing in the P10 BMSCs. In fact, long-term expansion in vitro will induce ageing of BMSCs [18, 22, 34]. In addition, our results showed that passage also had a slight effect on the immunomodulation capacity of BMSCs. Nevertheless, IR can decrease multiple cytokine secretion levels regardless of the P6 or P10 BMSCs. The immunomodulatory properties of mesenchymal stem cells are known to be important to their therapeutic applications [37]. Thus, assessing in vitro cell passage before application of BMSCs is important.

As reported, checkpoints can halt cell cycle progression temporarily in response to irradiation, thereby providing time for the repair of damaged DNA [38, 39]. Cell cycle arrest is only released following complete DNA repair, whereas cells with non-repairable DNA damage may undergo apoptosis [40]. Therefore, preferential activation of the DNA damage-induced checkpoint response may contribute to radio-resistance [41]. In our study, more noticeable S phase entry was observed in the P10 BMSCs after irradiation, suggesting exhaustion of the G1/S checkpoint in the P10 BMSCs. The increased number of cells in G2 phase in the P10 BMSCs after irradiation may result from a large number of cells with un-repaired DNA directly entering the S and then G2 phases. As expected, the percentage of apoptotic cells was increased to a greater extent in the P10 than in the P6 BMSCs at 24 and 48 h post-irradiation.

Irradiation of BMSCs may induce a premature cell cycle arrest or ageing (senescence) phenotype [38, 42]. In our study, the P10 BMSCs shared many gene expression characteristics that usually were induced by irradiation, such as senescence [13]. The transcriptional analysis revealed similarly persistent cell cycle arrest in both the P6 and P10 BMSCs at 2 h and 8 h post-irradiation, with altered expression of numerous cell cycle and oocyte meiosis genes. This characteristic pattern of “ageing” changes was previously detectible within 2 h following irradiation. Prior studies evaluated expression changes for select genes after irradiation in many cell lines and revealed a wave of differentially expressed genes modulating the G2/M transition of in mitotic cell cycle [43–48]. Thus, the signature of transcriptional changes that persisted in the irradiated BMSCs was similar to changes that occurred in BMSCs in the late passage during in vitro expansion.

Cell cycle-associated genes are broadly considered to regulate the radiation response [46, 49, 50]. This study provides the first genome-wide expression profile of irradiated BMSCs in different cell passages under in vitro expansion. Indeed, analysis of the BMSC transcriptomes demonstrated that some p53 signaling pathway genes were significantly upregulated at 2 h and 8 h post-irradiation in both the P6 and P10 BMSCs. For example, CDKN1A (P21), MDM2, GDF15 and GADD45 are important radiation response genes in the p53 signaling pathway [51–54]. Moreover, our network analysis identified other specific genes with potential regulatory roles. In our study, these genes showed a significant increase in expression after irradiation in both the P6 and P10 BMSCs. Thus, shared mechanisms most likely mediate the DNA damage and DNA repair in both the P6 and P10 BMSCs.

KEGG pathway analysis revealed that the upregulated genes in the P6 vs. P10 comparison were enriched in the cell cycle, which indicated high cell division activity. In particular, p21 showed a higher protein mRNA level in the P6 than P10 BMSCs, whereas the mRNA level showed reverse trend. Loss of p21 was reported to increase sensitivity to ionizing radiation [55]. Additionally, HJURP protein level was obviously decreased in the P6 but not in the P10 BMSCs at 2 h post-irradiation. In fact, breast cancer cells with high HJURP levels are more sensitive to radiation and exhibit a higher rate of apoptosis than those with low levels [56]. High HJURP expression may be one cause of cell cycle deregulation [57]. Therefore, further exploration of the mechanisms by which p21 and HJURP regulate differences in the response to irradiation between the two BMSC cell passages is necessary.

In addition, the upregulated genes in the P10 BMSCs were associated with some metabolic pathways and the PPAR signaling pathway. As reported, PPAR agonists can decrease osteogenesis and increase adipogenesis [58]. Moreover, our previous study demonstrated that Crif1 mediated the regulatory roles of PPARs in osteogenic and adipogenic differentiation of bone marrow MSCs after irradiation [25]. A recent report concluded that BMSCs at early passages must be used for osteogenic differentiation and that adipogenic potential might be better preserved over osteogenesis in aged BMSCs [22]. Radioresistance is different for BMSCs under various differentiation commitments [11]. The dysregulation of genes in the PPAR signaling pathway in the P10 BMSCs indicated that the radiation toxicity and response of BMSCs might depend on the cell passage. In other words, different molecular mechanisms mediate the irradiation tolerance for BMSCs at different cell passages from extensive in vitro expansion.

## Conclusion

In summary, in vitro cell passage affects the irradiation response of BMSCs by changing the gene expression profile and thus mediating variation in the cell cycle, apoptosis and senescence. We conclude that variation in the cell cycle distribution, ageing and differentiation potential during in vitro expansion plays a role in regulating radiation tolerance in BMSCs. Our results provide important cues for future studies on BMSCs from in vitro expansion and the application of BMSCs in therapy.

## Additional files

Additional file 1: **Table S1.** Primers that were used for RT-PCR in this study. (XLSX 10 kb)

Additional file 2: **Figure S1.** Cellular morphology and growth kinetics of BMSCs. **a** Cellular morphology of BMSCs from the passage P6 to P10. Scale bars = 100 μm. Growth kinetics of BMSCs from the passage P6 to P10 was induced by doubling time (DT) (**b)** in hours and population doubling (PD) (**c**). Data were expressed as mean ± SEM, *n* = 4. (TIF 1956 kb)

Additional file 3: **Figure S2.** Identification of BMSCs. **a** Typical surface marker for the P6 and P10 BMSCs was detected using flow cytometry. **b** The potential of osteogenic, chondrogenic and adipogenic differentiation of the P6 and P10 BMSCs was verified. (TIF 9629 kb)

Additional file 4: **Figure S3.** Senescence of BMSCs. Senescent cell was detected using β-Galactosidase Staining (**a**) and HP1-γ staining (**b**) in the P6 and P10 BMSCs with or without irradiation. (TIF 9479 kb)

Additional file 5: **Table S2.** Summary of sample names, description, the RNA-seq sequencing information and mapping results in each sample. Sample names were used directly in this study. Spliced reads mean reads spanning junctions. (XLSX 10 kb)

Additional file 6: **Table S3.** Human genes detection level by different RPKM cut off. (XLSX 8 kb)

Additional file 7: **Table S4.** Gene expression level (FPKM). (XLSX 2800 kb)

Additional file 8: **Figure S4.** Gene expression profiles of BMSCs before and after irradiation. **a** Unsupervised analysis of all genes that expressed at least in one sample. **b** The top10 enriched KEGG pathways for up (left) and down (right) regulated genes between the P6 and P10 BMSCs at 2 h post-irradiation. The numbers after each bar indicate the detected genes (left) and the total background genes involved in the pathway, respectively. **c** The top10 enriched KEGG pathways for up- (left) and downregulated (right) genes between the P6 and P10 BMSCs at 8 h post-irradiation. The numbers after each bar indicate the detected genes (left) and the total background genes involved in the pathway, respectively. **d** The top 10 enriched KEGG pathways for up- (left) and downregulated (right) genes between the P6 and P10 BMSCs common for three time points. The number after each bar indicate the detected genes (left) and the total background genes involved in the pathway, respectively. (TIF 2711 kb)

Additional file 9: **Table S5.** Differential expression of genes between P6 and P10 BMSCs at the same radiation time. (XLSX 956 kb)

Additional file 10: **Table S6.** Differential expression of genes between radiation times in the P6 or P10 BMSCs. (XLSX 756 kb)

Additional file 11: **Figure S5.** Functional analysis of the differential expression genes in BMSCs. The top10 enriched KEGG pathways of up- (a) or downregulated (b) genes between different time points shared by the P6 and P10 BMSCs. The top 10 most enriched Gene Ontology (GO) biology processes (BP) for genes up- (c) and downregulated (d) after irradiation. The colour scale shows the significance (*P* value) of pathways. (TIF 4355 kb)

Additional file 12: **Figure S6.** RT-PCR validation. a Relative expression level of radiation response genes measured by RNA-seq (FPKM). b Relative expression compared to P10_0h measured by qRT-PCR. (TIF 1758 kb)

Additional file 13: **Figure S7.** Protein expression levels of conserved irradiation response genes in BMSCs. **a** Representative immunofluorescence staining of CDKN1A, GDF15, and p53 in BMSCs. DAPI (blue), detected proteins (red) and merged images were shown. The photos were selected randomly. Scale bar 50 μm. **b** Quantification of immunofluorescence intensity; **c** Relative expression levels of protein normalized to actin are shown (see Fig. 4c for western blot results). (TIF 7330 kb)

Additional file 14: **Figure S8.** Weighted gene co-expression correlation network analysis (WGCNA) of differential expression genes. a Differentially expressed genes after irradiation (2 h vs. 0 h, 8 h vs. 0 h, 8 h vs. 2 h) in the P6 or P10 BMSCs were extracted to apply WGCNA analysis. b Heat map showing the co-expression modules by WGCNA. c Dendrogram from gene co-expression network analysis of samples from 0 h to 8 h time points. Modules of co-expressed genes were assigned colour. Correlations between gene co-expression modules. Module plots the top 15 hub genes and the top 50 connections along with the GO term enrichment of module MEturquoise (d), MEblack (e) and MEblue (f). The blue line represents negative correlation. The red line represents positive correlation. The size of point represents the number of genes associated with other genes. (TIF 2238 kb)

Additional file 15: **Table S7.** KEGG pathway enrichment for co-expression modules from WGCNA analysis. (XLSX 108 kb)

## Abbreviations

BMSC: Bone marrow mesenchymal stem cell
DEG: Differentially expressed genes
FDR: False discovery rate
FPKM: Fragments per kilobase of transcript per million fragments mapped
GO: Gene ontology
KEGG: Kyoto Encyclopedia of Genes and Genomes
PCA: Principal component analysis
ROS: Reactive oxygen species
WGCNA: Weighted gene co-expression network analysis
