# Supplementary figures and images for: In vitro expansion affects the response of human bone marrow stromal cells to irradiation
Source: Stem Cell Res Ther. 2019 Mar 8;10:82. doi: 10.1186/s13287-019-1191-3 (PMC6408817; doi:10.1186/s13287-019-1191-3)

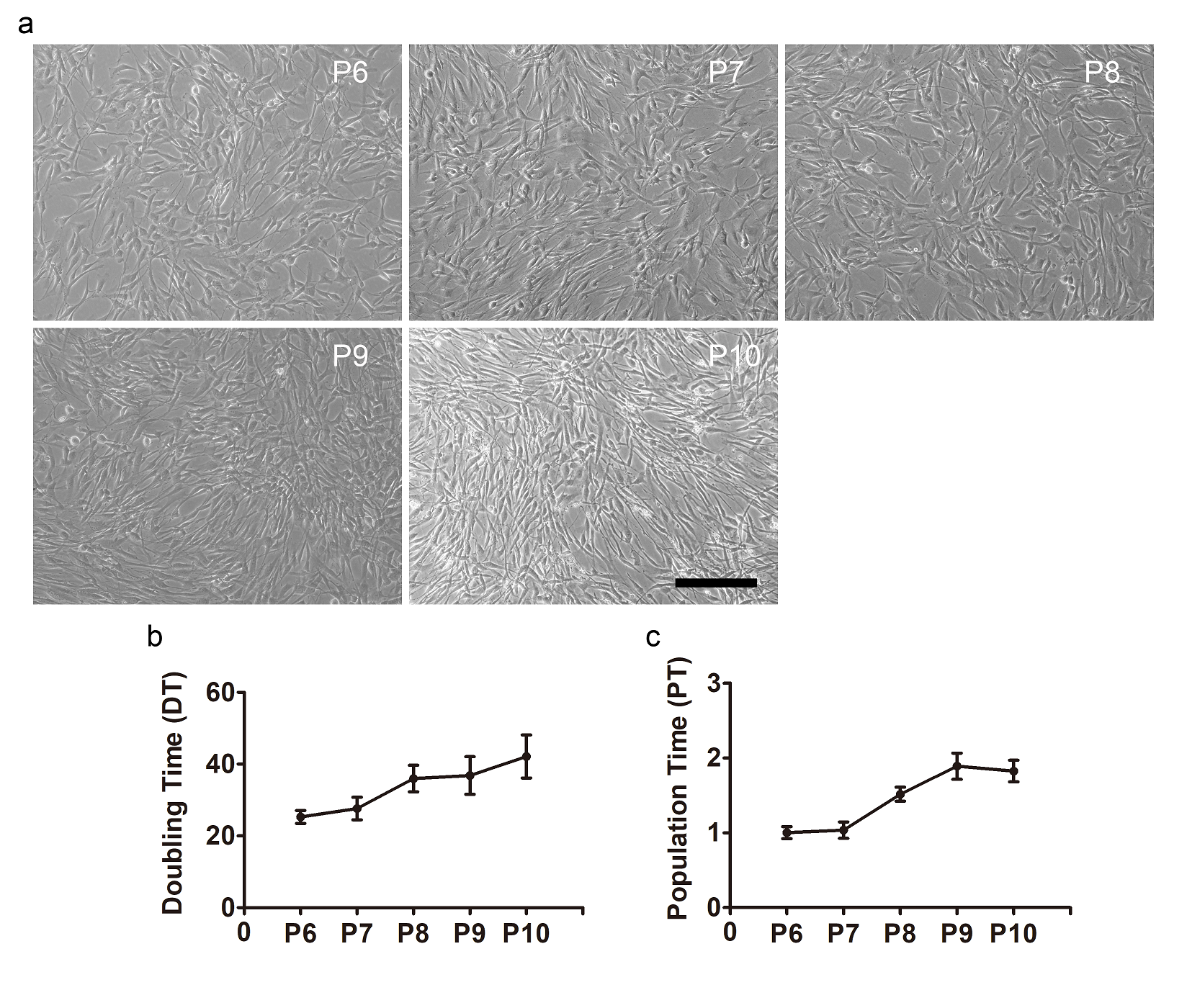

Supplement: Supplementary file 2 — Figure S1. Cellular morphology and growth kinetics of BMSCs. a Cellular morphology of BMSCs from the passage P6 to P10. Scale bars = 100 μm. Growth kinetics of BMSCs from the passage P6 to P10 was induced by doubling time (DT) (b) in hours and population doubling (PD) (c). Data were expressed as mean ± SEM, n = 4. (TIF 1956 kb) [file 13287_2019_1191_MOESM2_ESM.tif]

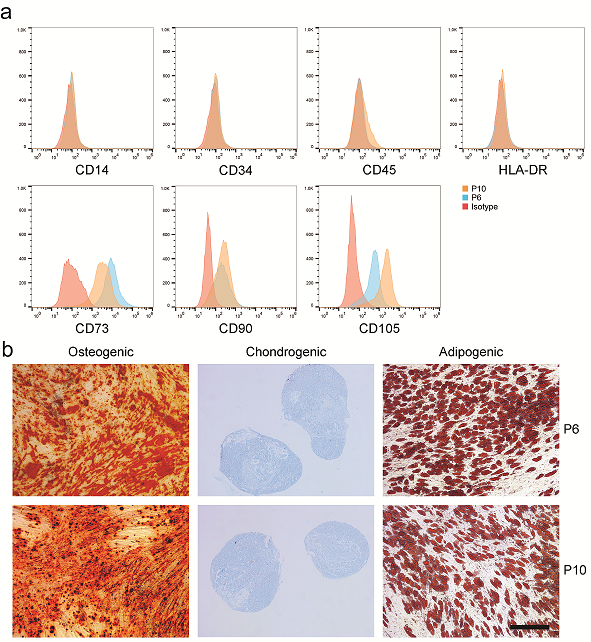

Supplement: Supplementary file 3 — Figure S2. Identification of BMSCs. a Typical surface marker for the P6 and P10 BMSCs was detected using flow cytometry. b The potential of osteogenic, chondrogenic and adipogenic differentiation of the P6 and P10 BMSCs was verified. (TIF 9629 kb) [file 13287_2019_1191_MOESM3_ESM.tif]

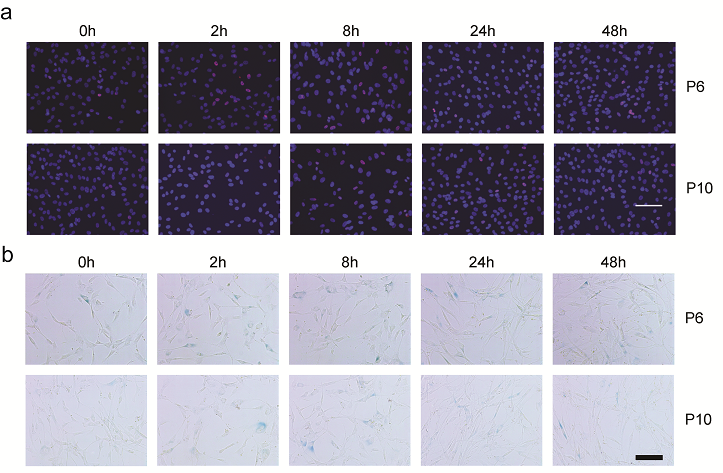

Supplement: Supplementary file 4 — Figure S3. Senescence of BMSCs. Senescent cell was detected using β-Galactosidase Staining (a) and HP1-γ staining (b) in the P6 and P10 BMSCs with or without irradiation. (TIF 9479 kb) [file 13287_2019_1191_MOESM4_ESM.tif]

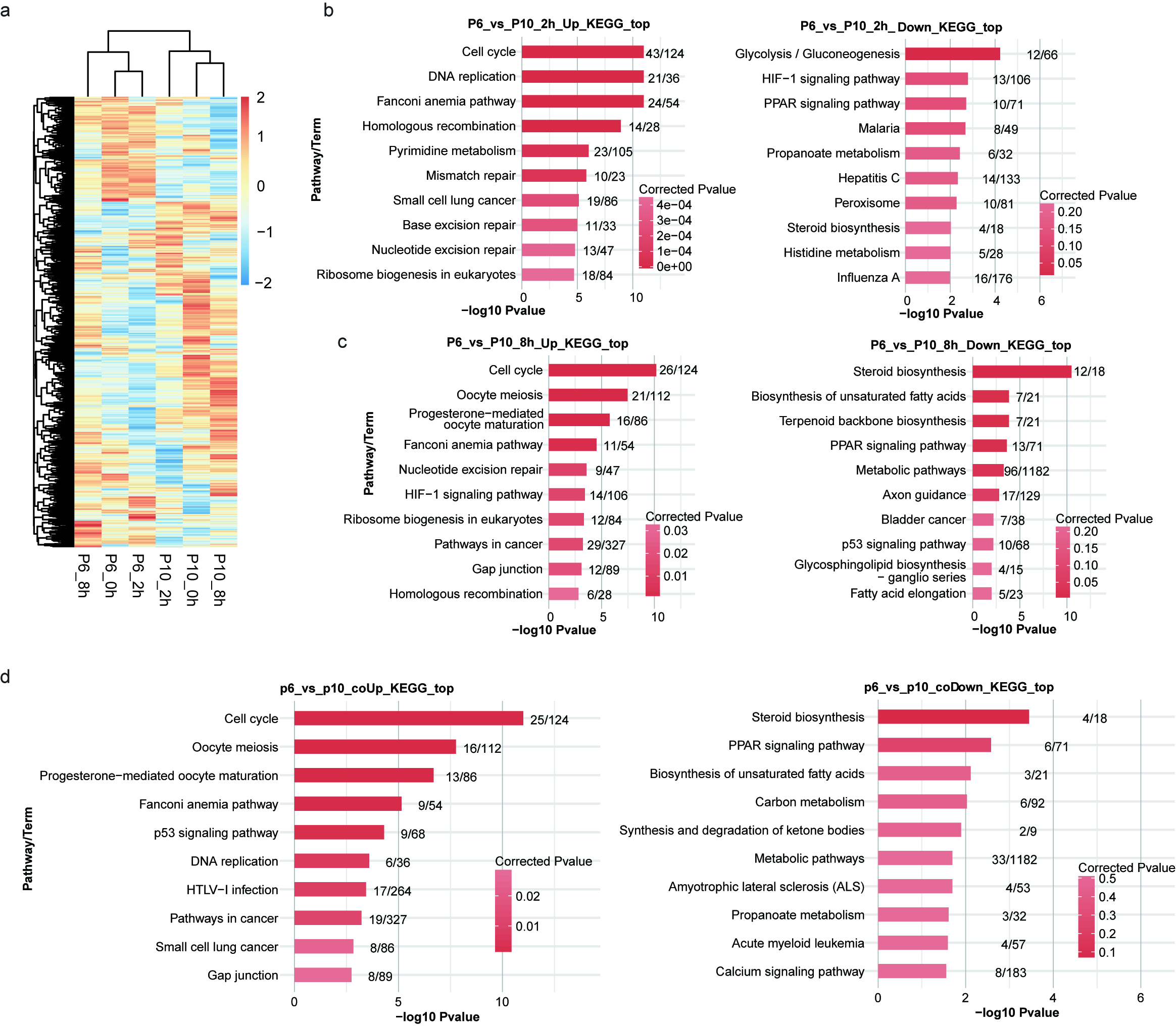

Supplement: Supplementary file 8 — Figure S4. Gene expression profiles of BMSCs before and after irradiation. a Unsupervised analysis of all genes that expressed at least in one sample. b The top10 enriched KEGG pathways for up (left) and down (right) regulated genes between the P6 and P10 BMSCs at 2 h post-irradiation. The numbers after each bar indicate the detected genes (left) and the total background genes involved in the pathway, respectively. c The top10 enriched KEGG pathways for up- (left) and downregulated (right) genes between the P6 and P10 BMSCs at 8 h post-irradiation. The numbers after each bar indicate the detected genes (left) and the total background genes involved in the pathway, respectively. d The top 10 enriched KEGG pathways for up- (left) and downregulated (right) genes between the P6 and P10 BMSCs common for three time points. The number after each bar indicate the detected genes (left) and the total background genes involved in the pathway, respectively. (TIF 2711 kb) [file 13287_2019_1191_MOESM8_ESM.tif]

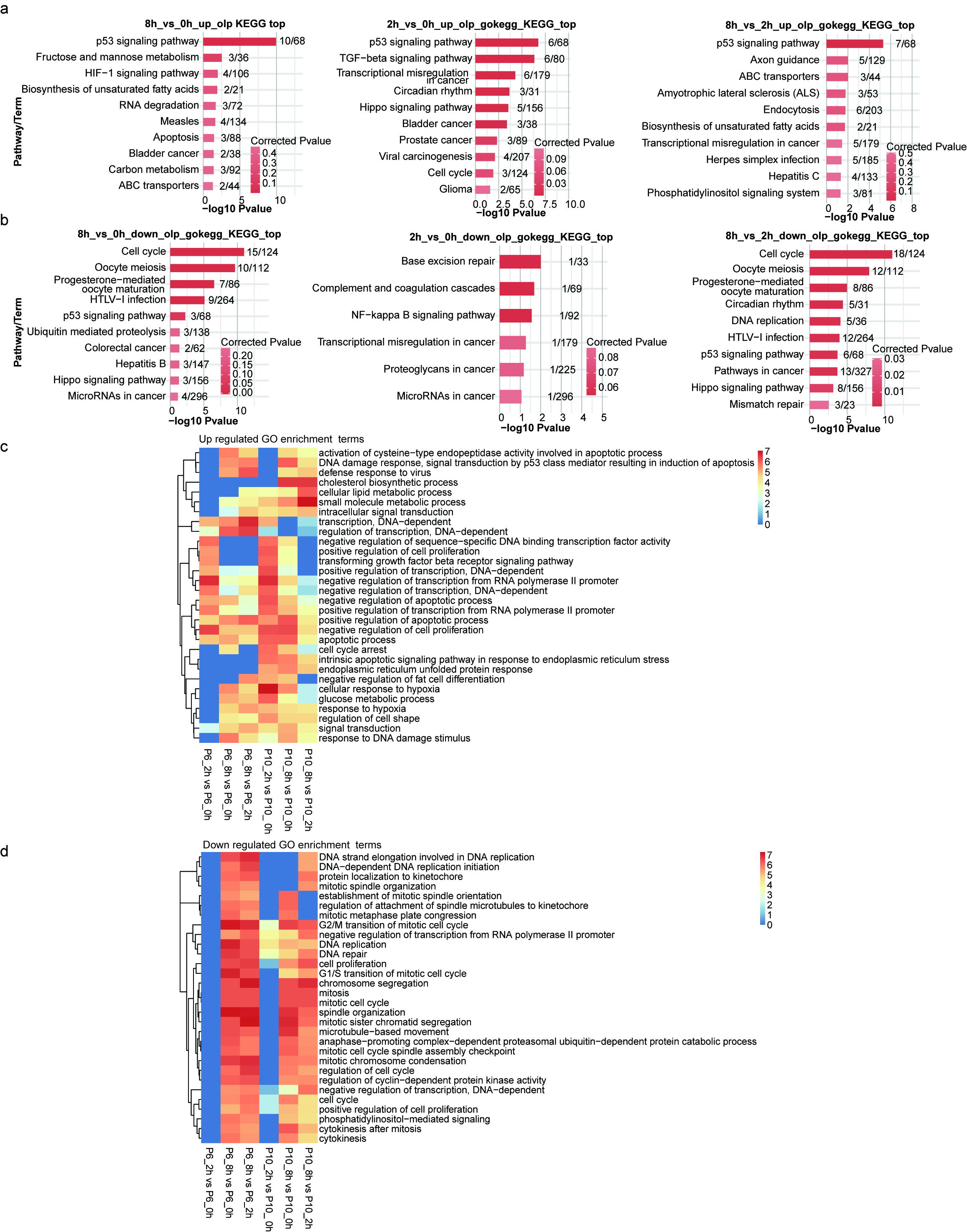

Supplement: Supplementary file 11 — Figure S5. Functional analysis of the differential expression genes in BMSCs. The top10 enriched KEGG pathways of up- (a) or downregulated (b) genes between different time points shared by the P6 and P10 BMSCs. The top 10 most enriched Gene Ontology (GO) biology processes (BP) for genes up- (c) and downregulated (d) after irradiation. The colour scale shows the significance (P value) of pathways. (TIF 4355 kb) [file 13287_2019_1191_MOESM11_ESM.tif]

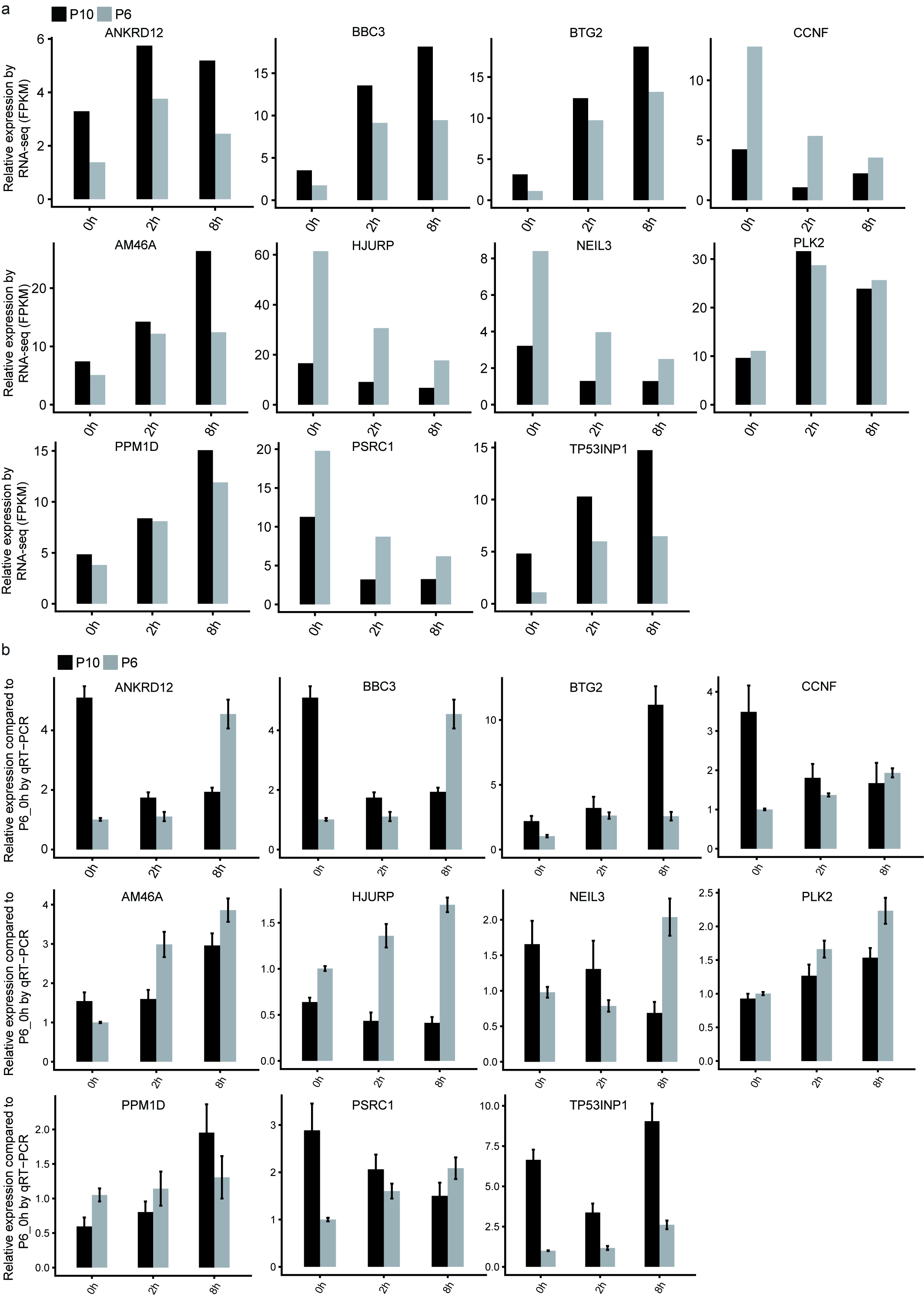

Supplement: Supplementary file 12 — Figure S6. RT-PCR validation. a Relative expression level of radiation response genes measured by RNA-seq (FPKM). b Relative expression compared to P10_0h measured by qRT-PCR. (TIF 1758 kb) [file 13287_2019_1191_MOESM12_ESM.tif]

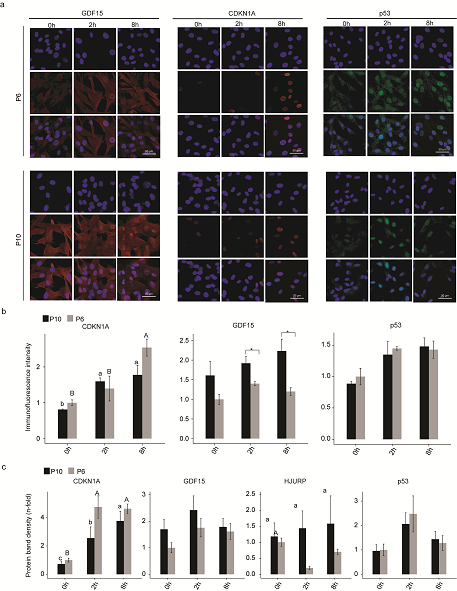

Supplement: Supplementary file 13 — Figure S7. Protein expression levels of conserved irradiation response genes in BMSCs. a Representative immunofluorescence staining of CDKN1A, GDF15, and p53 in BMSCs. DAPI (blue), detected proteins (red) and merged images were shown. The photos were selected randomly. Scale bar 50 μm. b Quantification of immunofluorescence intensity; c Relative expression levels of protein normalized to actin are shown (see Fig. 4c for western blot results). (TIF 7330 kb) [file 13287_2019_1191_MOESM13_ESM.tif]

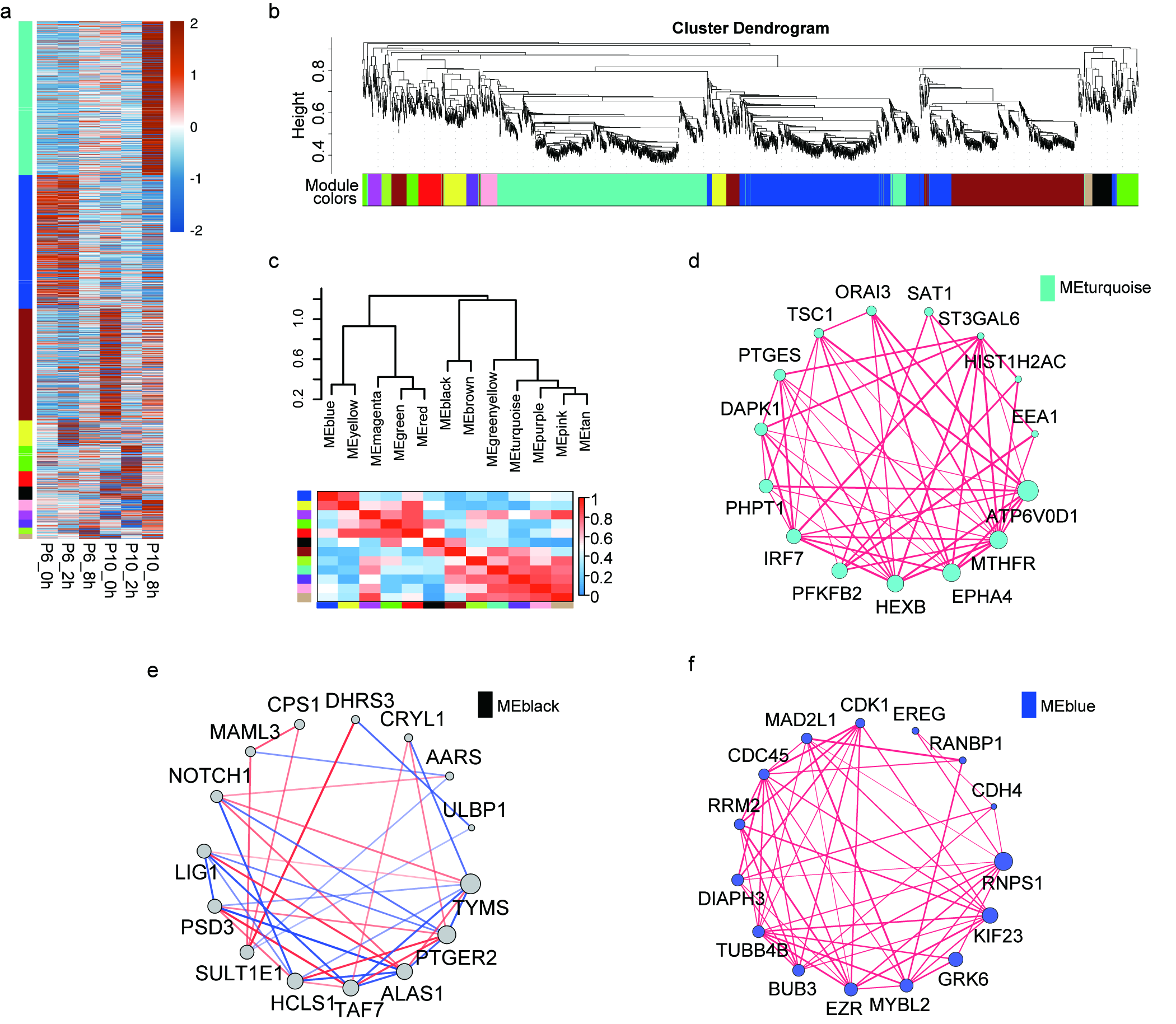

Supplement: Supplementary file 14 — Figure S8. Weighted gene co-expression correlation network analysis (WGCNA) of differential expression genes. a Differentially expressed genes after irradiation (2 h vs. 0 h, 8 h vs. 0 h, 8 h vs. 2 h) in the P6 or P10 BMSCs were extracted to apply WGCNA analysis. b Heat map showing the co-expression modules by WGCNA. c Dendrogram from gene co-expression network analysis of samples from 0 h to 8 h time points. Modules of co-expressed genes were assigned colour. Correlations between gene co-expression modules. Module plots the top 15 hub genes and the top 50 connections along with the GO term enrichment of module MEturquoise (d), MEblack (e) and MEblue (f). The blue line represents negative correlation. The red line represents positive correlation. The size of point represents the number of genes associated with other genes. (TIF 2238 kb) [file 13287_2019_1191_MOESM14_ESM.tif]
